# Relationship between serum LDH levels and diabetic peripheral neuropathy in type 2 diabetic patients

**DOI:** 10.3389/fendo.2025.1680539

**Published:** 2025-10-29

**Authors:** Meng Sun, Zhaodi Wang, Xu Yan, Hangyu Shen, Hongqiao Yang, Yuxia Qi, Xiang Gao, Yi Huang, Jie Sun

**Affiliations:** ^1^ Ningbo Key Laboratory of Nervous System and Brain Function, Department of Neurosurgery, The First Affiliated Hospital of Ningbo University, Ningbo, Zhejiang, China; ^2^ Department of Neurosurgery, The First Affiliated Hospital of Shandong First Medical University & Shandong Provincial Qianfoshan Hospital, Jinan, Shandong, China; ^3^ Department of Internal Medicine, Qingdao Public Health Clinical Center, Qingdao, Shandong, China

**Keywords:** type 2 diabetes mellitus, diabetic peripheral neuropathy, lactate dehydrogenase, biomarker, risk assessment

## Abstract

**Background:**

Diabetic peripheral neuropathy (DPN) is commonly observed as a long-term complication in patients with type 2 diabetes mellitus (T2DM). Recent evidence suggests that metabolic disturbances and chronic inflammation may contribute to its development. Lactate dehydrogenase (LDH), a key enzyme in glycolysis, may reflect underlying metabolic stress and inflammation, but its association with DPN remains unclear.

**Methods:**

In this cross-sectional study, 2,060 patients with T2DM were analyzed to explore the relationship between serum LDH levels and DPN. Logistic regression and restricted cubic spline (RCS) models were used to assess linear and non-linear associations. Participants were also stratified by age, sex, hypertension, and HbA1c levels for subgroup analyses.

**Results:**

Among the study population, 724 (35.1%) had DPN. Higher LDH levels were independently associated with an increased risk of DPN after adjusting for potential confounders (adjusted OR per 1 U/L increase: 1.00; 95% CI: 1.00–1.01; P = 0.01). RCS analysis showed a non-linear relationship, with a threshold at 142 U/L. Participants with LDH >142 U/L had significantly higher odds of DPN (adjusted OR: 1.21; 95% CI: 1.02–1.48; P = 0.033). This association was consistent across subgroups.

**Conclusion:**

Serum LDH levels are significantly and non-linearly associated with DPN in individuals with T2DM. LDH may serve as a simple and cost-effective biomarker for identifying patients at elevated risk of neuropathy, warranting further prospective validation.

## 1 Introduction

The global prevalence of diabetes mellitus (DM) has been rising at an alarming rate, posing a substantial public health burden. As of 2021, approximately 537 million adults were living with diabetes, and this number is projected to reach 783 million by 2045 (1). Among its many complications, diabetic peripheral neuropathy (DPN) is one of the most common and debilitating microvascular consequences, affecting nearly half of individuals with type 2 diabetes mellitus (T2DM) (2–4). DPN not only leads to sensory and motor impairments but also increases the risk of diabetic foot ulcers, infections, non-traumatic amputations, falls, and mortality (5–7).

DPN is characterized by a chronic, progressive course with often irreversible nerve damage. Despite its clinical significance, the pathophysiological mechanisms underlying DPN remain incompletely understood. Accumulating evidence suggests that hyperglycemia-induced metabolic disturbances—including oxidative stress, mitochondrial dysfunction, inflammation, and microvascular insufficiency—play central roles in the development and progression of DPN (8, 9). Among these factors, oxidative stress is considered a key driver of peripheral nerve injury, as excessive reactive oxygen species (ROS) can impair both endothelial function and neuronal integrity (10, 11).

Lactate dehydrogenase (LDH), a key enzyme in glycolysis that catalyzes the interconversion of pyruvate and lactate, has recently attracted attention as a potential biomarker of metabolic stress and tissue injury. Under normal physiological conditions, LDH is primarily confined within cells, but its serum concentration increases with cell membrane damage and tissue hypoxia (12). Experimental studies have shown that hyperglycemia and hypoxia can elevate LDH levels in retinal cells, highlighting its possible involvement in diabetic microvascular complications (13).

In clinical contexts, elevated LDH has been associated with adverse outcomes in various diseases characterized by hypoxia, inflammation, and oxidative stress, including cardiovascular disease, malignancies, and diabetic nephropathy (14–16). Given these associations and the biological plausibility linking LDH to oxidative and metabolic stress, LDH may also serve as a systemic marker for DPN risk. However, few studies have systematically examined the association between serum LDH levels and the risk of DPN.

Therefore, this study aimed to investigate the association between serum LDH concentrations and the presence of DPN in a nationally representative cohort of individuals with T2DM. Additionally, we applied restricted cubic spline (RCS) modeling to explore potential non-linear relationships and identify clinically relevant threshold effects, with the goal of evaluating the potential utility of LDH as a biomarker for diabetic neuropathy.

## 2 Material and methods

### 2.1 Data source

**Figure 1:**
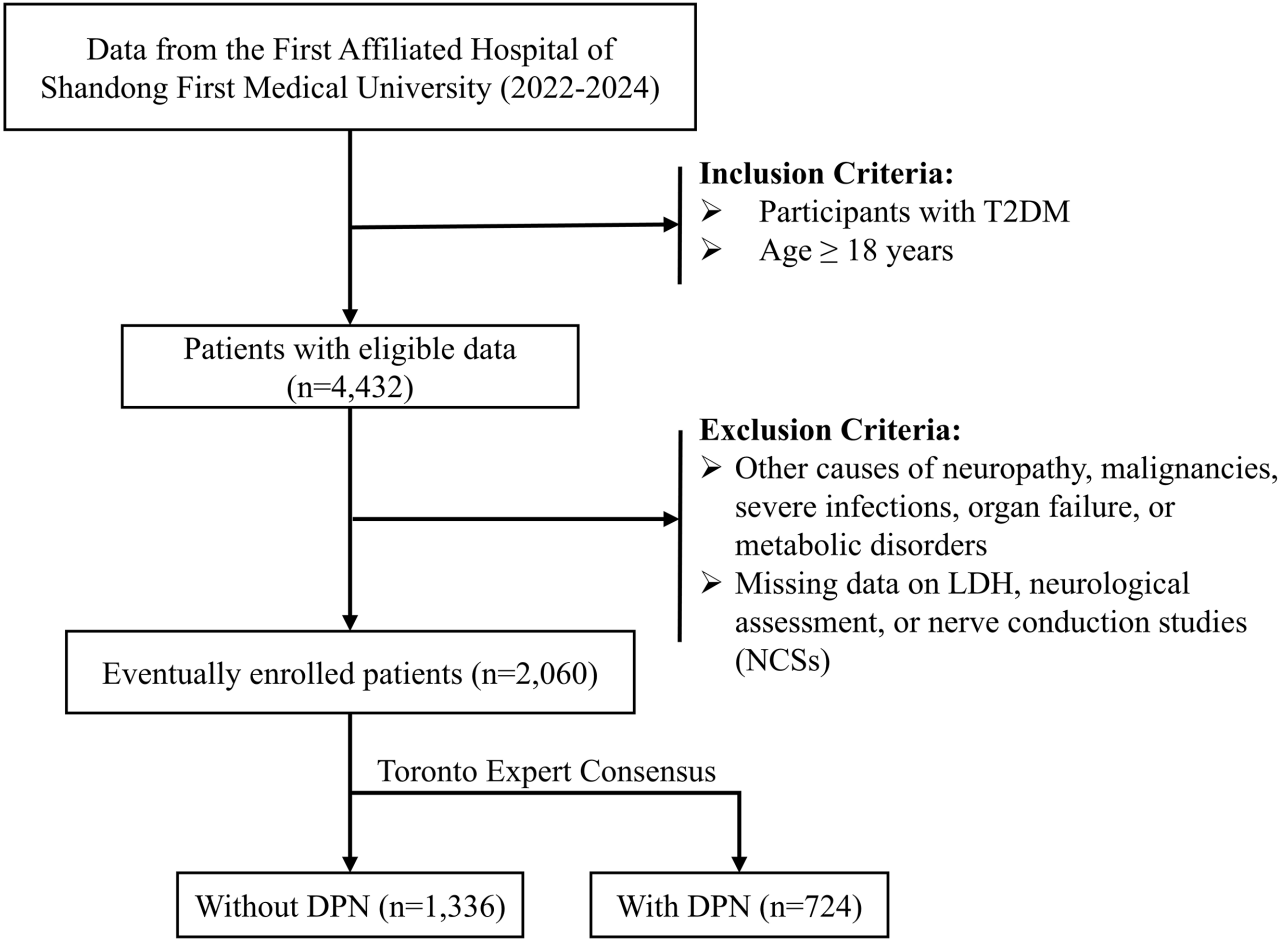
Flow chart of the selection process.

This retrospective study was carried out at the First Affiliated Hospital of Shandong First Medical University, including patients treated between January 2022 and December 2024. The inclusion criteria were as follows (1): patients aged 18 years or older; and (2) a diagnosis of T2DM established according to the 1999 World Health Organization (WHO) criteria. The exclusion criteria were as follows (1): neuropathy due to other identifiable causes, such as malignancies, severe infections, organ failure, or metabolic disorders; and (2) missing information on serum LDH, neurological assessment, or nerve conduction studies (NCSs). A detailed flowchart of the participant selection process is presented in **Figure 1**. This study was approved by the Ethics Committee of the First Affiliated Hospital of Shandong First Medical University (Approval No. S654) and conducted in accordance with the principles of the Declaration of Helsinki.

### 2.2 Covariates

In this study, a range of covariates potentially associated with DPN were collected and included in the analysis. Demographic and lifestyle factors consisted of sex, age, body mass index (BMI), smoking status, and alcohol consumption history. Laboratory parameters encompassed hematological indices such as white blood cell count (WBC), lymphocyte count (Lymph), monocyte count (Mono), neutrophil count (Neut), platelet count (PLT), and hemoglobin (Hb). Liver and kidney function indicators included alanine aminotransferase (ALT), aspartate aminotransferase (AST), total bilirubin (TBil), serum creatinine (SCR), albumin (ALB), globulin (GLB), urea, and uric acid (UA). Lipid profile variables comprised total cholesterol (TC), triglycerides (TG), high-density lipoprotein cholesterol (HDL-C), and low-density lipoprotein cholesterol (LDL-C). In addition, lactate dehydrogenase (LDH), alkaline phosphatase (ALP), glycated hemoglobin (HbA1c), and diabetes duration were also recorded as covariates.

### 2.3 Statistical analysis

Descriptive statistics for the continuous and categorical variables were reported in the form of means, SDs, and frequencies, respectively. Group comparisons were performed using the t-test for continuous variables and the chi-square test for categorical variables.

Logistic regression models were applied to evaluate the association between LDH levels and the risk of DPN, with results presented as odds ratios (ORs) and 95% confidence intervals (CIs). Model 1 was unadjusted. Model 2 was adjusted for age, sex, BMI, smoking status, and alcohol consumption history. Model 3 was fully adjusted, incorporating all potential confounders, including sex, age, BMI, smoking status, alcohol consumption history, WBC, Lymph, Mono, Neut, PLT, Hb, ALT, AST, TBil, SCR, ALB, GLB, urea, UA, TC, TG, HDL-C, LDL-C, ALP, HbA1c, and diabetes duration.

To assess the potential association between LDH and DPN, a restricted cubic spline (RCS) analysis was conducted based on complex sampling logistic regression. Subgroup analyses and interaction tests were performed to explore effect modification by sex, age (<65 vs. ≥65 years), hypertension, and HbA1c level (<7% vs. ≥7%). Heterogeneity across subgroups was evaluated using multivariable logistic regression models including interaction terms. All statistical analyses were conducted using R software (version 4.4.2). A two-sided P-value < 0.05 was considered statistically significant.

## 3 Result

### 3.1 Baseline characteristics

**Table 1** summarizes the baseline characteristics of the 2,060 patients with T2DM included in this study, among whom 724 (35.1%) were diagnosed with DPN. The overall mean age was 60.58 (11.70) years, and 1,057 (51.3%) were female. The mean serum LDH level was 146.16 U/L. Compared to patients without DPN, those with DPN were significantly older and had a higher proportion of hypertension and smoking history (all P < 0.05). In terms of laboratory parameters, patients with DPN exhibited significantly lower levels of Lymph, Hb, ALB, and TC, while showing elevated levels of Neut, TBil, SCR, LDH, UA, and longer duration of diabetes (all P < 0.05).

**Table 1:** Comparison of baseline characteristics between DPN and non-DPN patients.

Characteristics	Overall (n = 2,060)	Non-DPN (n = 1,336)	DPN (n = 724)	*P* value
Age, years	64.58 (11.70)	62.71 (11.42)	68.03 (11.42)	<0.001
Sex, % Male Female	1,003 (48.7) 1,057 (51.3)	647 (48.4) 689 (51.6)	356 (49.2) 368 (50.8)	0.783
BMI, % Normal Overweight Obesity	736 (35.7) 991 (48.1) 333 (16.2)	483 (36.2) 639 (47.8) 214 (16.0)	253 (34.9) 352 (48.6) 119 (16.4)	0.859
Hypertension, % No Yes	750 (36.4) 1310 (63.6)	528 (39.5) 808 (60.5)	222 (30.7) 502 (69.3)	<0.001
Smoking, % No Yes	1,409 (68.4) 651 (31.6)	938 (70.2) 398 (29.8)	471 (65.1) 253 (34.9)	0.019
Drinking, % No Yes	1,224 (59.4) 836 (40.6)	791 (59.2) 545 (40.8)	433 (59.8) 291 (40.2)	0.828
WBC, × 10^9^/L	7.50 (2.15)	7.48 (2.10)	7.55 (2.25)	0.439
Lymph, × 10^9^/L	2.18 (0.89)	2.24 (0.88)	2.06 (0.90)	<0.001
Mono, × 10^9^/L	0.52 (0.18)	0.52 (0.18)	0.54 (0.18)	0.147
Neut, × 10^9^/L	4.49 (1.68)	4.41 (1.57)	4.63 (1.85)	0.005
PLT, × 10^9^/L	224.31 (62.18)	226.13 (63.04)	220.96 (60.46)	0.072
Hb, g/L	141.31 (15.82)	142.07 (15.41)	139.93 (16.54)	0.004
ALT, U/L	26.75 (19.79)	27.30 (19.48)	25.72 (20.32)	0.082
AST, U/L	25.58 (15.56)	25.56 (15.70)	25.60 (15.32)	0.952
TBil, μmol/L	10.99 (4.75)	10.82 (4.68)	11.29 (4.85)	0.032
SCR, μmol/L	87.40 (71.99)	76.28 (35.58)	107.91 (108.50)	<0.001
ALB, g/L	41.97 (3.46)	42.27 (3.35)	41.43 (3.59)	<0.001
GLB, g/L	31.96 (4.96)	32.02 (4.77)	31.85 (5.31)	0.474
Urea, mmol/L	5.78 (2.57)	5.73 (2.60)	5.86 (2.52)	0.273
UA, μmol/L	335.10 (93.01)	328.61 (92.84)	347.07 (92.21)	<0.001
TC, mmol/L	5.22 (1.20)	5.31 (1.22)	5.05 (1.14)	<0.001
TG, mmol/L	2.30 (2.41)	2.36 (2.54)	2.20 (2.16)	0.171
HDL-C, mmol/L	1.00 (0.23)	1.00 (0.24)	1.00 (0.23)	0.73
LDL-C, mmol/L	2.17 (0.90)	2.14 (0.88)	2.21 (0.94)	0.128
LDH, U/L	146.16 (36.51)	144.04 (34.78)	150.06 (39.22)	<0.001
ALP, U/L	82.73 (30.65)	82.87 (31.38)	82.47 (29.27)	0.776
HbA1c, %	7.70 (1.79)	7.49 (1.79)	8.10 (1.72)	<0.001
Diabetes duration, years	6.10 (11.67)	4.90 (10.28)	8.31 (13.60)	<0.001

Data are mean (standard deviation), or n (%).DPN indicates diabetic peripheral neuropathy; BMI, body mass index; WBC, white blood cell count; Lymph, lymphocyte count; Mono, monocyte count; Neut, neutrophil count; PLT, platelet count; Hb, hemoglobin; ALT, alanine aminotransferase; AST, aspartate aminotransferase; SCR, serum creatinine; ALB, albumin; GLB, globulin; Urea, urea; UA, uric acid; TC, total cholesterol; TG, triglycerides; HDL-C, high-density lipoprotein cholesterol; LDL-C, low-density lipoprotein cholesterol; LDH, lactate dehydrogenase; ALP, alkaline phosphatase.

### 3.2 Association between LDH and DPN

**Figure 2:**
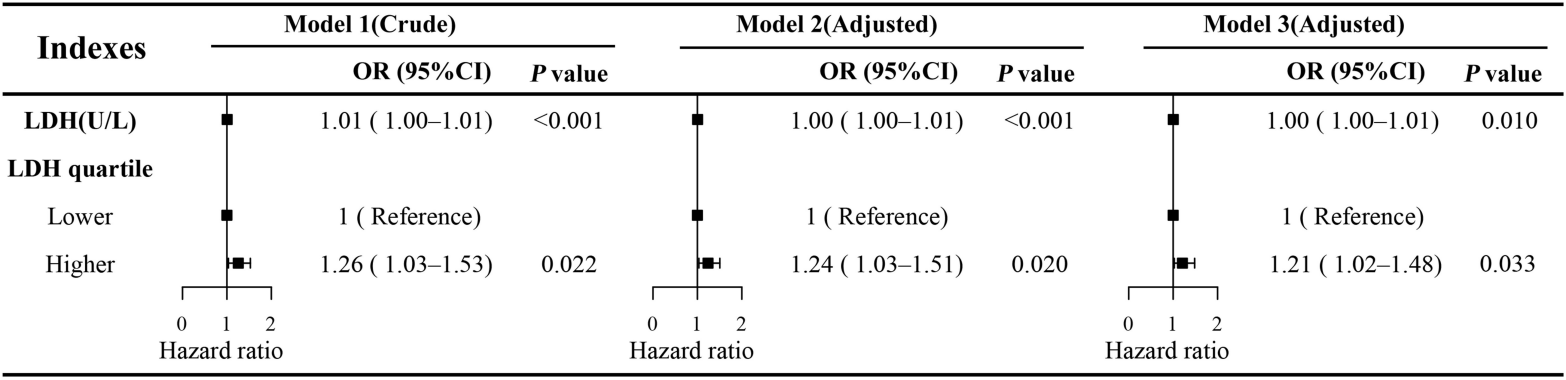
Multivariate logistic regression models of LDH and DPN. Model 1 was unadjusted. Model 2 was adjusted for age, sex, BMI, smoking status, and alcohol consumption history. Model 3 was fully adjusted for all potential confounders, including age, sex, BMI, smoking status, alcohol consumption history, white blood cell count (WBC), lymphocyte count (Lymph), monocyte count (Mono), neutrophil count (Neut), platelet count (PLT), hemoglobin (Hb), alanine aminotransferase (ALT), aspartate aminotransferase (AST), total bilirubin (TBil), serum creatinine (SCR), albumin (ALB), globulin (GLB), urea, uric acid (UA), total cholesterol (TC), triglycerides (TG), high-density lipoprotein cholesterol (HDL-C), low-density lipoprotein cholesterol (LDL-C), alkaline phosphatase (ALP), glycated hemoglobin (HbA1c), and diabetes duration. OR, odds ratio; CI, confidence interval; LDH, lactate dehydrogenase; DPN, diabetic peripheral neuropathy.

The results of the univariate logistic regression analysis revealed a significant association between serum LDH levels and the risk of DPN. Specifically, each 1 U/L increase in LDH was associated with a higher risk of DPN (OR: 1.01; 95% CI: 1.00–1.01; P < 0.001) (**Figure 2**). This association remained robust after adjusting for potential confounding factors. In Model 2, which adjusted for age, sex, body mass index (BMI), smoking status, and alcohol consumption, the association persisted (OR: 1.00; 95% CI: 1.00–1.01; P < 0.001). In the fully adjusted Model 3, which further included hypertension, HbA1c levels, and other relevant laboratory parameters, LDH remained independently associated with DPN risk (OR: 1.00; 95% CI: 1.00–1.01; P = 0.01). Although the effect size per unit increase was small, given the broad distribution of LDH levels in the population, this cumulative effect may be of clinical significance.

**Figure 3:**
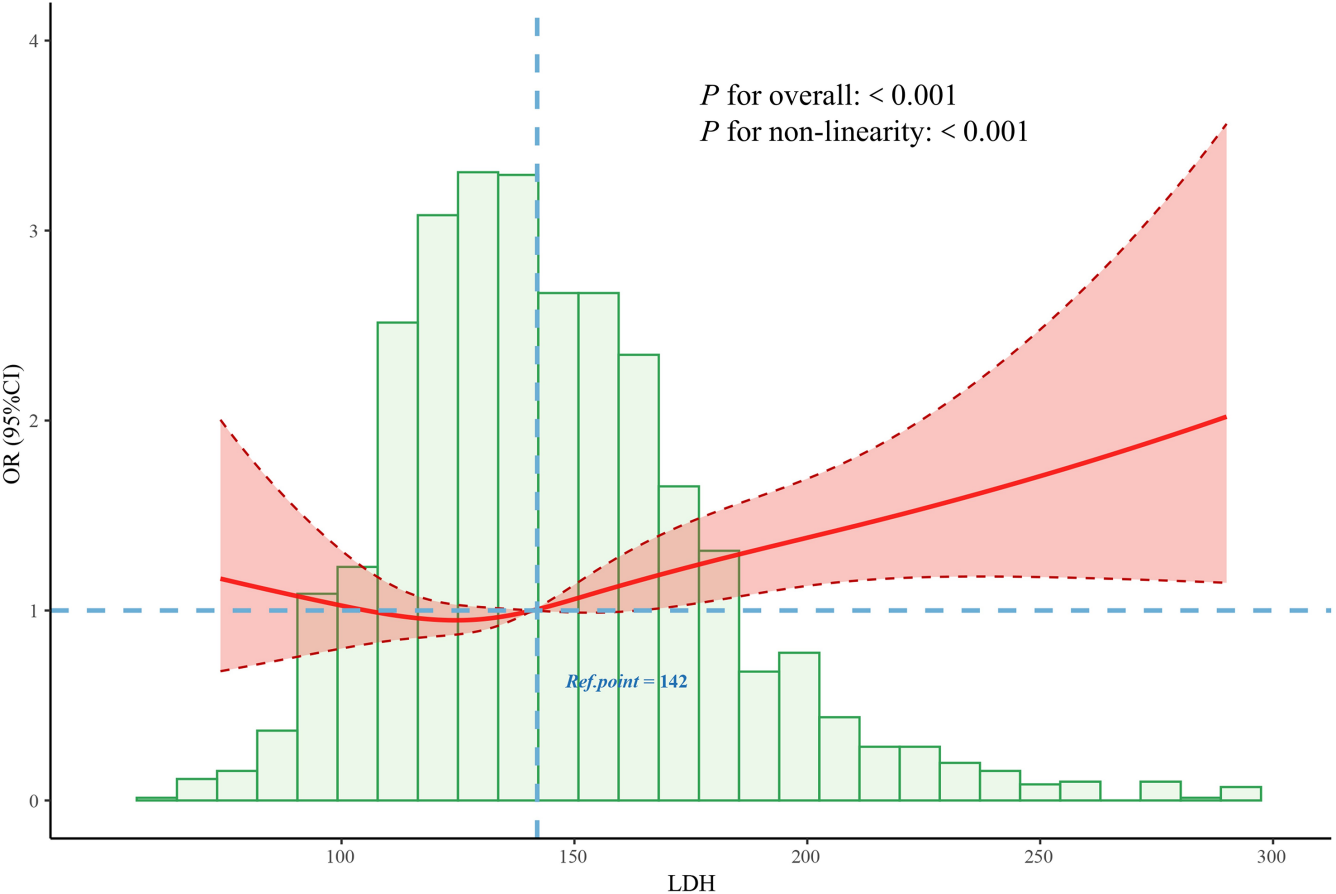
Association between the LDH and DPN odds ratio. The solid line represents the estimated odds ratio, and the shaded area indicates the 95% confidence interval. LDH, lactate dehydrogenase; DPN, diabetic peripheral neuropathy.

To further explore the dose–response relationship, the RCS regression model was applied (**Figure 3**). The RCS analysis revealed a significant non-linear association between continuous LDH levels and DPN risk (P for non-linearity < 0.001), with an inflection point observed at approximately 142 U/L. Based on this threshold, LDH was subsequently categorized into two groups: low LDH (≤142 U/L) and high LDH (>142 U/L). Multivariate logistic regression analysis demonstrated that individuals in the high LDH group had a significantly greater risk of developing DPN compared to those in the low LDH group (OR: 1.21; 95% CI: 1.02–1.48; P = 0.033) (**Figure 2**).

**Figure 4:**
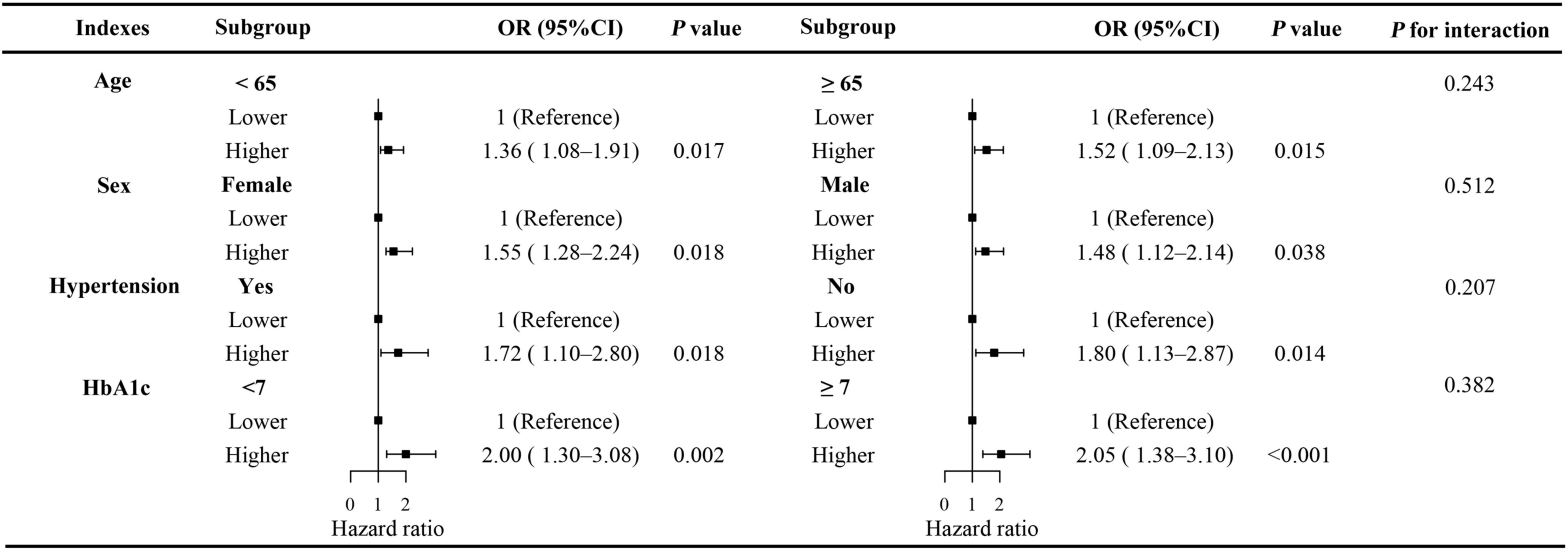
Stratified analysis of the association between serum LDH levels and the risk of DPN across subgroups. Subgroup analyses were performed based on age (<65 vs. ≥65 years), sex (male vs. female), hypertension (yes vs. no), and HbA1c (<7.0% vs. ≥7.0%). LDH, lactate dehydrogenase; DPN, diabetic peripheral neuropathy; OR, odds ratio; CI, confidence interval.

Stratified analyses showed that the association between LDH levels and the risk of DPN remained consistent across subgroups defined by age (<65 vs. ≥65 years), sex (male vs. female), hypertension (yes vs. no), and HbA1c (<7.0% vs. ≥7.0%) (**Figure 4**). No significant interaction was observed in any subgroup (all P for interaction > 0.05).

## 4 Discussion

In this cross-sectional study, we found that higher serum LDH levels were independently associated with an increased risk of DPN in individuals with T2DM. This association persisted after controlling for multiple confounders, and a non-linear relationship was observed using RCS analysis, with a turning point at 142 U/L. Participants with LDH levels >142 U/L exhibited significantly higher odds of DPN compared to those with LDH ≤142 U/L. These findings were consistent across subgroups stratified by age, sex, hypertension status, and HbA1c level, with no significant interaction effects detected.

In our univariate analysis, DPN was associated with several clinical and biochemical abnormalities. Patients with DPN were older and more likely to have hypertension and a history of smoking, suggesting the cumulative impact of aging and vascular comorbidities on neural degeneration in diabetes (17, 18). Consistent with previous studies, higher HbA1c levels and longer diabetes duration were observed in the DPN group, highlighting the role of chronic hyperglycemia in exacerbating peripheral nerve damage (19, 20). Furthermore, DPN patients exhibited lower lymphocyte counts, hemoglobin, albumin, and total cholesterol levels, alongside elevated neutrophil counts, bilirubin, serum creatinine, uric acid, and LDH, which may reflect a systemic milieu of inflammation, oxidative stress, nutritional deficiency, and subclinical organ dysfunction—all contributing factors in the pathogenesis of DPN (19, 21).

Although LDH is not traditionally recognized as a biomarker for DPN, several biological mechanisms may underlie the observed association. LDH is a key enzyme in anaerobic glycolysis, and elevated LDH may reflect increased anaerobic metabolism, mitochondrial dysfunction, or systemic metabolic stress (22, 23). In the context of diabetes, chronic hyperglycemia induces oxidative stress, promotes the formation of advanced glycation end-products (AGEs), and triggers inflammatory responses, all of which contribute to peripheral nerve damage (24). Moreover, LDH is released during tissue injury and inflammatory processes. Recent studies suggest that low-grade systemic inflammation plays a pivotal role in the development of DPN by activating immune cells, promoting endothelial dysfunction, and impairing microvascular circulation to peripheral nerves (25–30). Therefore, elevated LDH levels may serve as a surrogate marker for ongoing subclinical inflammation and neuronal damage.

To date, few studies have explored the relationship between LDH and DPN specifically. However, elevated LDH has been associated with diabetic complications such as nephropathy and retinopathy (13, 16), which share common pathogenic mechanisms with DPN, including microvascular dysfunction and chronic inflammation. Our study adds novel evidence to this limited body of research by demonstrating a significant, dose-dependent relationship between LDH and DPN using both linear and non-linear modeling approaches.

Importantly, LDH is a routinely tested, low-cost biomarker in clinical settings. The identification of a specific threshold (142 U/L) linked to increased DPN risk offers practical value for early screening and risk stratification in diabetic patients. Individuals with elevated LDH may benefit from closer clinical monitoring, comprehensive metabolic assessment, and early preventive interventions targeting neuropathy.

Several limitations should be acknowledged. First, the cross-sectional design precludes conclusions about causality or the temporal sequence between elevated LDH and DPN onset. Second, although we adjusted for a wide range of potential confounders, residual confounding from unmeasured variables (e.g., medications, infections, or unrecorded comorbidities) cannot be excluded. Third, LDH levels were measured at a single time point, which may not fully reflect long-term metabolic or inflammatory status.

## 5 Conclusion

In conclusion, our findings reveal a significant and non-linear association between serum LDH levels and the risk of DPN in patients with T2DM. These results suggest that LDH may serve as an accessible biomarker for identifying individuals at higher risk of neuropathy. Future prospective studies and experimental investigations are warranted to validate these findings and to explore the underlying biological pathways linking LDH to peripheral nerve injury.

## Data Availability

The raw data supporting the conclusions of this article will be made available by the authors, without undue reservation.
